# Radiological Features in Type II Odontoid Fractures in Older Adults After High- and Low-Energy Trauma

**DOI:** 10.1177/21925682221088215

**Published:** 2022-03-28

**Authors:** Pavlina Lenga, Mohammed Issa, Lennart Krull, Karl Kiening, Andreas W. Unterberg, Till Schneider, Rod J. Oskouian, Jens R. Chapman, Basem Ishak

**Affiliations:** 1 Department of Neurosurgery, 9144 Heidelberg University Hospital, Heidelberg, Germany; 2 Department of Neuroradiology, 570672 Heidelberg University Hospital, Heidelberg, Germany; 3 214855 Division of Complex Spine Swedish Neuroscience Institute, Seattle, WA, USA

**Keywords:** cervical spine, elderly, trauma, type II odontoid fractures, low-energy trauma, high-energy trauma, cervical malalignment, osteoporosis

## Abstract

**Study design:**

Retrospective study.

**Objectives:**

Although type II odontoid fractures mainly occur due to high-energy trauma (HET), the number of odontoid type II fractures after low-energy trauma (LET) in the elderly is on the rise. However, there is a paucity of conclusive evidence on the relationship between trauma mechanism and cervical spine alignment in the elderly population. Consequently, we examined cervical alignment and osteoporotic and osteoarthritic patterns in elderly individuals (aged ≥65 years) with type II odontoid fractures.

**Methods:**

We retrospectively assessed cervical spine alignment in 76 elderly individuals who experienced type II odontoid fractures after HET (n = 36) and LET (n = 40) between 2005 and 2020. Osteoporotic and osteoarthritic changes on computed tomography and cervical alignment parameters on sagittal plane radiographs were examined.

**Results:**

Moderate and severe osteoporosis of the dens-body junction and osteoarthritis of the atlanto-odontoid joint were more prevalent in the LET than the HET group (*P*<.005). The anterior atlantodental interval (ADI) was significantly smaller in the LET group than in the HET group (.7 [.7] millimeter vs 1.2 [.8] mm; *P*=.003). An ADI equal 0 mm indicative for anterior fusion of C1/C2 was present in 37.5% of patients of the LET group. The C0-C2 angle, C1-C2 lordosis, and C2-C7 sagittal vertical axis were significantly different (HET vs LET: 33.2 [7.2]° vs 41.6 [11.4]°, *P*=.005; 28.1 [7.0]° vs 34.0 [8.0]°, *P*=.002; and 16.1 [11.1] millimeter vs 27.1 [12.4] mm, *P*=.008; respectively).

**Conclusion:**

Significantly higher rates of osteoporotic and degenerative changes were observed after LET. Furthermore, previous cervical malalignment represents a risk factor for type II odontoid fractures after LET.

## Introduction

With the global trend of increasing life expectancy due to accelerating improvements in the quality of health care worldwide, geriatric trauma has been gaining increasing attention. Type II odontoid fractures are among the most common cervical spine fractures in older adults, comprising 40-82% of odontoid fractures.^1^ In older adults, low-energy trauma (LET) mechanisms such as falls from standing or seated positions mostly cause such fractures.^2-4^ In young individuals, however, they mainly occur due to high-energy trauma (HET) mechanisms such as traffic accidents or falls from height.

Injuries in older adults present a unique challenge because age-related degeneration is associated with bone density loss and stiffness of the middle and lower cervical spines. These factors contribute to a shift of the motion segment to the upper cervical spine, which is responsible for the higher prevalence of upper cervical spine fractures in older individuals.^2,5-7^ Osteoarthritic changes in the atlantoaxial joint may also increase the probability of older individuals sustaining type II odontoid fractures.^8^ However, no systematic analysis of the relationship between trauma mechanism and cervical spine alignment and degeneration has been conducted yet. Additionally, no study has described the different degenerative patterns in older individuals with type II odontoid fractures. Indeed, detailed evidence on type II odontoid fractures and cervical degeneration and malalignment is needed.

Therefore, this study is aimed to assess and compare radiological features and patterns of craniocervical degeneration and osteoporosis in older patients who have sustained type II odontoid fractures after LET and HET.

## Methods

### Study Design and Data Collection

We retrospectively evaluated the radiological data collected from the database of our institution between September 2005 and December 2020. The local ethics committee approved this study and waived the requirement for informed consent due to the retrospective nature of the study. Patients aged ≥65 years with type II odontoid fractures, diagnosed on both cervical spine X-ray and computed tomography (CT) images, were included. The exclusion criteria were congenital instability, rheumatoid arthritis, instability caused by a tumor, spinal infections, or previous cervical surgery. Patients’ injuries were classified as HET or LET according to the German guidelines for trauma mechanisms.^8^ HET was defined as a fall from a height >1 m or >5 steps, axial compression injury, or traffic accident at a speed ≥100 km/h, with rollover or ejection from the vehicle, or two-wheel/quad accident, or collision with a heavy vehicle (i.e., bus and truck). LET was defined as a fall from a sitting or standing position or a low height (<1 m).

### Imaging Analysis

Sagittal plane radiographs and cervical CT-scans were obtained from patients on admission. A collar was not used during the examinations. All angles of cervical alignment were measured with the fracture, including the dislocation of the odontoid. As part of the clinical routine at our institution, a standard X-ray was performed in anteroposterior and lateral views to evaluate the cervical spine alignment parameters. All CT studies were conducted using thin-layer CT. The measurements were performed by 2 experienced neurosurgeons (MI, LK); an additional measurement was performed by trained neuroradiologists (TS) to evaluate interobserver variability.

### Osteoporotic and Osteoarthritic Patterns

Osteoporotic changes in the cervical spine were examined on CT images using the technique proposed by Watanabe et al.^9^ We classified the osteoporosis severity of the dens-body junction and odontoid process as “none” (normal trabecular pattern with normal cortical thickness), “mild” (decrease in the number of trabeculae without areas of absent trabeculae [holes] and normal cortical thickness), “moderate” (absent trabeculae [holes] involving 50% of the transverse diameter of the bone with cortical thinning), or “severe” (absent trabeculae [holes] involving more than 50% of the transverse diameter of the bone with cortical thinning). The severity of osteoarthritis in the atlanto-odontoid, atlantoaxial, and atlanto-occipital joints was classified as “none” (normal joint space without osteophyte formation), “mild” (narrowed joint space or normal joint space with osteophyte formation), “moderate” (obliterated joint space with/without osteophyte formation), or “severe” (joint ankylosis with bony excrescences in the joint, transverse ligament calcification, or both).^10^

### Radiological Parameters of Cervical Alignment

Cervical alignment was assessed with CT images. The displacement of the odontoid fracture was determined by drawing lines along the posterior aspect of the dens ligament and the intact caudal body of C2. The odontoid process height was defined as the length of the vertical line from the apex of the odontoid process to the dento-central synchondrosis. The pB-C2 line was defined as the perpendicular distance between the spinal dura mater and the line connecting the basion to the inferior posterior edge of C2 in the median sagittal plane. The odontoid-McGregor distance was defined as the length of the line connecting the posterior edge of the hard palate and the most caudal point of the occipital curve. The odontoid-Chamberlain distance was defined as the length of the line connecting the posterior edge of the hard palate and the opisthion. The odontoid-McRae distance was defined as the length of the line connecting the basion and the opisthion. The anterior atlantodental interval (ADI) was defined as the distance between the posterior margin of the anterior arch of the atlas and the odontoid process. The posterior ADI was defined as the distance between the anterior margin of the posterior arch of the atlas and the odontoid process. An ADI equal to 0 mm was indicating the occurrence of fusion.

**Figure 1.**
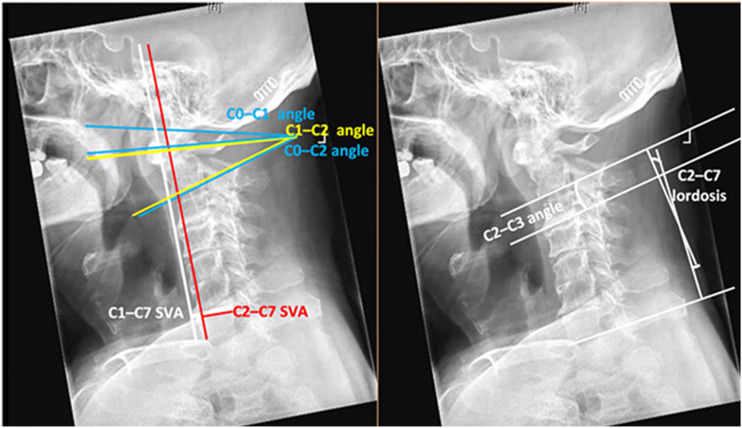
Sagittal radiographs showing the parameters for cervical alignment.

The following parameters were measured by examining sagittal plane radiographs (Figure 1): C0-C1 angle (formed by the McRae line and the line connecting the centers of the anterior and posterior arches of C1); C0-C2 angle (formed by the McRae line and the inferior endplate of C2); C1-C2 lordosis (angle formed by the line connecting the anterior tubercle to the spinous process of C1 and the inferior endplate of C2); C2-C3 lordosis (angle of intersection of the tangents to the inferior endplates of C2 and C3); C2-C7 lordosis (angle of intersection of the tangents to the inferior endplates of C2 and C7); C1-C7 sagittal vertical axis (SVA: distance between the plumb line extending from the anterior arch of C1 to the superior posterior edge of the endplate of C7); and C2-C7 SVA (distance between the plumb line extending from the centroid of C2 to the superior posterior edge of the endplate of C7).

### Statistical Analysis

Categorical variables are presented as numbers and percentages. Continuous variables are presented as means and standard deviations (SD); all were normally distributed (verified with the Shapiro-Wilk test). The baseline characteristics of the 2 groups were compared using independent t-tests for continuous variables and chi-squared tests for categorical variables. Fleiss’ kappa analysis was performed to evaluate the interobserver variability. The kappa values were interpreted as follows: ≤ .2 = slight agreement, .21-.4 = fair agreement, .41-.6 = moderate agreement, .61-.8 = substantial agreement, .81-.99 = almost perfect agreement, and 1.0 = perfect agreement.^11^ Cohen’s D analysis was performed to measure the effect size. A sample size of 30 cases per group is considered sufficient to describe the empirical distribution of the parameters and generate a hypothesis about systematic differences between the groups. Statistical significance was set at *P* < .05. All data were statistically evaluated by an external, independent statistician. All statistical analyses were performed using SPSS software (version 24.0.0.0; IBM, Armonk, NY, USA).

## Results

### Demographic Characteristics

The analysis included 40 and 36 older individuals who sustained type II odontoid fractures in the LET and HET groups, respectively, between September 2005 and December 2020. The overall mean age was 79.6 years; there was no significant difference in age between HET and LET groups (79.6 ± 6.1 vs 80.3 ± 6.8 years; *P* = .134). A male predominance in the HET group (21/36; 68.3%) and a slight female predominance in the LET group (21/40; 52.5%) were noted; however, this difference was not statistically significant (*P* = .345). Almost perfect or perfect interobserver agreement was observed for degeneration patterns and alignment parameters (Table 1). With this number of cases, a standardized effect size (Cohen’s D) of .74 with a power of 80% was obtained by an independent t-test with a 5% significance level.

**Table 1.** Interobserver agreement in the evaluation of osteoporotic and osteoarthritic patterns and radiological parameters.

	Fleiss’ Kappa (95% Confidence Interval)	*P*
Osteoporosis of the dens-body junction	.907 (.853-.943)	<.001
Osteoporosis of the odontoid process	.957 (.932-.973)	<.001
Osteoarthritis of the atlanto-odontoid joint	.982 (.972-.989)	<.001
Osteoarthritis of the lateral atlantoaxial joint	.922 (.886-.975)	<.001
Osteoarthritis of the atlanto-occipital joint	.982 (.972-.989)	<.001
Odontoid process height	.986 (.977-.991)	<.001
pB-C2 distance (mm)	.963 (.940-.977)	<.001
Odontoid-McGregor distance (mm)	.999 (.999-1.000)	<.001
Odontoid-Chamberlain distance (mm)	1.000 (1.000-1.000)	<.001
Odontoid-McRae distance (mm)	.966 (.945-.979)	<.001
Anterior atlantodental interval (mm)	.951 (.922-.969)	<.001
Posterior atlantodental interval (mm)	.993 (.990-.996)	<.001
C0-C1 angle (°)	.999 (.999-1.000)	<.001
C0-C2 angle (°)	.656 (.544-.788)	<.001
C1-C2 lordosis (°)	.997 (.996-.998)	<.001
C2-C3 lordosis (°)	.997 (.996-.998)	<.001
C2-C7 lordosis (degrees)	.996 (.993-.997)	<.001
C1-C7 sagittal vertical axis (°)	1.000 (1.000-1.000)	<.001
C2-C7 sagittal vertical axis (°)	1.000 (1.000-1.000)	<.001

### Degeneration Patterns

**Figure 2.**
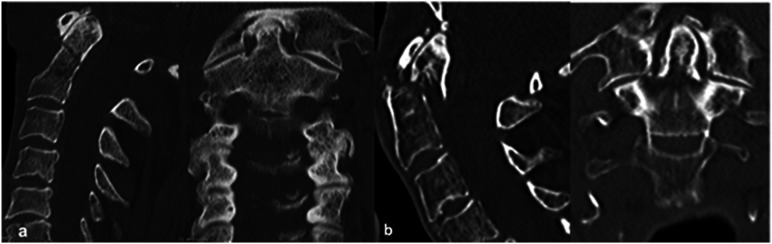
Sagittal and coronal computed tomography images of patients with type II odontoid fracture following (a) high-energy trauma and (b) low-energy trauma, depicting severe osteoporosis at the dens-body junction and no osteoarthritis in the lateral atlantoaxial joint.

Osteoporotic and degenerative patterns are shown in Table 2. The osteoporosis grade in the dens-body junction and odontoid process differed significantly between the 2 groups (*P* < .05; Table 2, Figure 2). The prevalence of moderate and severe degeneration of the atlanto-odontoid joint was higher in the LET group (27/40, 67.5%) than in the HET group (14/36 33.3%; *P* < .001).

**Table 2.** Comparison of osteoporotic and osteoarthritic patterns between the low- and high-energy trauma groups.

	High-energy trauma n = 36	Low-energy trauma n = 40	*P*
Osteoporosis of the dens-body junction			**.003**
None	2 (5.6)	2 (5.0)	
Mild	18 (50.0)	6 (15.0)	
Moderate	14 (38.9)	25 (62.5)	
Severe	2 (5.6)	7 (17.5)	
Osteoporosis of the odontoid process			**<.001**
None	4 (11.1)	2 (5.0)	
Mild	18 (50.0)	4 (10.0)	
Moderate	13 (36.1)	26 (65.0)	
Severe	1 (2.8)	8 (20.0)	
Osteoarthritis of the atlanto-odontoid joint			**<.001**
None	5 (13.9)	2 (5.0)	
Mild	19 (52.8)	11 (27.5)	
Moderate	11 (30.6)	14 (35.0)	
Severe	1 (2.8)	13 (32.5)	
Osteoarthritis of the lateral atlantoaxial joint			**.046**
None	17 (47.2)	8 (20.0)	
Mild	15 (41.7)	26 (65.0)	
Moderate	4 (11.1)	4 (10.0)	
Severe	0 (.0)	2 (5.0)	
Osteoarthritis of the atlanto-occipital joint			**.002**
None	13 (36.1)	3 (7.5)	
Mild	21 (58.3)	29 (72.5)	
Moderate	2 (5.6)	7 (17.5)	
Severe	0 (.0)	0 (.0)	

Data are presented as numbers (percentages). The highlighted *P*-values indicate statistically significant results.

**Figure 3.**
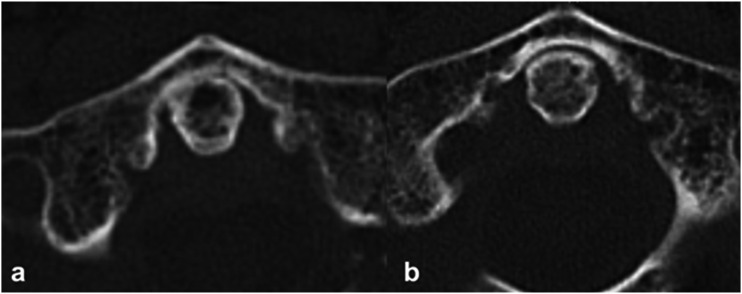
Axial computed tomography reconstruction showing (a) severe degeneration of the atlanto-odontoid joint in patients with low-energy trauma and (b) absence of degeneration in patients with high-energy trauma.

Figure 3 displays severe degeneration of the atlanto-odontoid joint in patients with LET fractures and its absence in patients with HET fractures.

### Radiological Features of Cervical Alignment

On initial assessment, 67 patients (88.2%) had a posterior odontoid fracture displacement, while only 9 patients (11.8%) presented with an anterior one. Overall the mean was 1.5 mm (SD 1.7 mm). No significant differences were obtained between the groups concerning the severity of displacement (HET 1.5 mm SD 1.7 vs LET 1.3 mm SD 1.7; *P* = .806). The anterior ADI was equal 0 mm, indicative of a fusion of the anterior arch with the dens, in 8.3% of patients from the HET group (n = 3/36) and in 37.5% of patients from LET group (n = 15/40). The range of anterior ADI in the HET group was 0-4.5 mm and of the LET group was 0-2.3 mm. The anterior ADI was significantly different between the 2 groups (LET: .7 ± .7 mm vs HET: 1.2 ± .8 mm; *P* = .003). No significant differences were observed between the groups concerning the posterior ADI (LET: 20.5 ± 3.5 mm vs HET: 20.8 ± 2.9 mm; *P* = .6). The C0-C2 angle and C1-C2 lordosis were significantly higher in the LET group (41.6 ± 11.4° and 34.0 ± 8.0°, respectively) than in the HET group (33.2 ± 7.2° and 28.1 ± 7.0°; *P* = .005 and *P* = .002, respectively). A significant difference in the C2-C7 SVA was observed between the groups (LET: 27.1 ± 12.4 mm vs HET: 16.1 ± 11.1 mm; *P* = .008). Other differences between groups were non-significant. Table 3 shows the radiological parameters of cervical alignment in the LET and HET groups.

**Table 3.** Comparison of radiological parameters of cervical alignment between the low- and high-energy trauma groups.

	High-energy trauma n = 36	Low-energy trauma n = 40	*P*
Odontoid process height (mm)	25.2 (2.4)	26.2 (1.8)	.194
pB-C2 (mm)	4.3 (1.6)	4.2 (2.5)	.681
Odontoid-McGregor distance (mm)	.06 (3.3)	−.33 (3.7)	.402
Odontoid-Chamberlain distance (mm)	.6 (3.5)	.2 (3.4)	.384
Odontoid-McRae distance (mm)	4.4 (2.1)	4.7 (2.1)	.746
Anterior atlantodental interval (mm)	1.2 (.8)	.7 (.7)	**.003**
Posterior atlantodental interval (mm)	20.8 (2.9)	20.5 (3.5)	.600
C0-C1 angle (degrees)	5.1 (6.4)	7.8 (6.6)	.555
C0-C2 angle (degrees)	33.2 (7.2)	41.6 (11.4)	**.005**
C1-C2 lordosis (degrees)	28.14 (7.0)	34.0 (8.0)	**.002**
C2–C3 lordosis (degrees)	4.5 (6.9)	5.2 (7.6)	.166
C2-C7 lordosis (degrees)	12.4 (11.7)	11.4 (19.0)	.977
C1-C7 sagittal vertical axis (degrees)	22.1 (13.2)	34.4 (16.5)	.097
C2-C7 sagittal vertical axis (degrees)	16.1 (11.1)	27.1 (12.4)	**.008**

Data are presented as means (standard deviations). The highlighted *P*-values indicate statistically significant results.

### Discussion

Type II odontoid fractures are the most common fractures of the cervical spine in the elderly.^1^ While the mobile elderly population is rising steadily, the prevalence of LET most commonly produce type II odontoid fractures, which are challenging to treat. It is well known that due to degenerative changes of the cervical spine and owing to a substantial loss of bone density in the elderly, the body’s ability to absorb the energy of a trauma is significantly reduced. Hence, this patient cohort is at higher risk of sustaining an odontoid type II fracture even after a LET compared to younger patients.^2,5-7^ It is important to understand that osteoarthritic changes in the atlantoaxial joint as well as cervical malalignment might constitute critical factors that predispose the elderly to experiencing an odontoid type II fracture.^8^ However, strong evidence for a relationship between radiological features of the cervical spine and various injury mechanisms (HET vs LET) among the elderly with type II odontoid fractures is still scarce.

To our knowledge, this is the first systematic analysis to thoroughly describe trauma mechanisms in older individuals sustaining type II odontoid fractures and changes in cervical alignment. The current study examined degeneration patterns exclusively in older individuals who had sustained a type II odontoid fracture after HET and LET to determine possible predictors of this type of fracture. Severe and moderate osteoporosis of the dens-body junction and odontoid process was significantly more common in patients with LET than in those with HET, findings which are comparable to previously published reports.^9,11^ In addition, severe and moderate osteoarthritis of the dens-body and atlanto-odontoid junction occurred more frequently in patients with LET than those with HET, as previously described.^9^ Severe and moderate degeneration of the atlanto-odontoid joint were observed in 67.5% of the elderly with LET in this study. In contrast, only 33.3% of the cases showed atlanto-odontoid joint degeneration after HET. Furthermore, only C2-C7 SVA, C0-C2, and C1-C2 angles surpassed the established normal values after LET as determined by Patel et al,^12^ while no substantial changes were observed after HET. In line with previous studies, one might consider these values as indicators for performing surgery.^13^ Furthermore, the mean anterior ADI in the LET group was substantially lower,^14^ while the ADI was normal in the HET group. Interestingly, there was an almost perfect or perfect agreement for all the examined radiological features. It bears noting that the effect size of our results was remarkably high at .74.

#### Literature

Recently, there has been a steady increase in the prevalence and incidence of odontoid type II fractures compared to other spinal injuries in elderly patients.^17^ Cervical spinal degeneration is age-dependent and more prevalent in individuals older than 40 years.^18,19^ Hence, these degenerative processes in the cervical spine substantially influence the susceptibility of the elderly to sustain fractures of the atlantoaxial complex, especially the odontoid.^9,11^ Watanabe et al,^9^ in their retrospective analysis of 21 older patients, showed that both moderate and severe osteoporosis was frequently associated with type II odontoid fractures after LET; however, they did not examine HET cases. In another study on degeneration patterns after LET with concomitant type II odontoid fracture,^10^ degeneration of the atlanto-odontoid joint was strikingly high at 90.5%, which may be explained by the fact that the image quality of CT scanners in the 2000s was inferior to that of scanners used nowadays. In the aforementioned studies, the rate of lateral atlantoaxial joint degeneration was low or it was even absent. This might support the view that, due to the presence of a degenerative and fixed atlanto-odontoid joint in conjunction with a smooth lateral atlantoaxial joint, older individual are more prone to sustaining type II odontoid fractures after a simple fall since an axial rotation of the head induces consistent torque forces on the osteoporotic dens-body, which represents the rotary center. Therefore, degenerative processes, solely in LET, may play a crucial role in the occurrence of type II odontoid fractures.

The cervical spine is the most mobile part of the spine, with the upper and lower cervical spine having different ranges of motion, which are affected by increasing age and concomitant degeneration. Liu et al.^14^ in their retrospective analysis of ADI as examined on CT scans found a mean ADI of 1.07 mm in individuals older than 70 years. Specifically, the authors stated that anterior ADI depends exclusively on age because in this subset of patients, the degeneration of the atlanto-odontoid joint, ligament classification and the higher rates of osteoarthritis contribute to a substantial decrease of the anterior ADI with values between 0 mm and 2.3 mm. That phenomenon was also reflected in our results, since we found that approximately 50% of our cohort had an anterior ADI of 0 mm, thus indicating a fused atlanto-odontoid joint. While larger anterior ADIs are considered a paramount parameter to diagnose dislocation of the atlanto-odontoid joint in young patients,^15^ in the elderly, due to the degenerative processes it seems that already smaller anterior ADIs may facilitate substantial dislocation of the atlanto-odontoid joint. Furthermore, previous evidence support that a posterior ADI below 14 mm might be correlated with the occurrence of neurological deficits as narrowing of the posterior ADI can cause compression of vascular structures such as the anterior spinal artery or direct compression of the dural sack.^16^ However, in our study the values of the posterior ADI were consistently in a normal range for both HET and LET groups; thus indicating that the posterior ADI might play a secondary role when evaluating odontoid fractures in the elderly. Consequently, when examining dislocation of odontoid fractures in this subset of patients, anterior ADI should be considered an important parameter for determining subluxation or dislocation of the middle atlantodental joint*.*

With the advent of advanced imaging techniques, physicians are now able to quantify spinal alignment in a patient by using a myriad of different parametric measurements of the cervical, thoracic, and lumbosacral spine. Nonetheless, meticulous examination of cervical malalignment after traumatic injuries in the elderly has so far only played a marginal role in the literature. In a retrospective study on surgical techniques in 46 patients who had sustained a type II odontoid fracture, a C0-C1 angle >6.7°, a C1-C2 angle >19.1°, and a C2-C7 angle >19.6° were indicators for surgery.^13^ Notably, Oshima et al^13^ examined a wider age range of 15-81 years and did not focus on elderly patients, thus explaining some differences in their values. It seems that in cases of type II odontoid fractures in the elderly population, surgical correction of cervical malalignment should be considered after meticulous study of cervical alignment parameters.

As previously described, in older patients, posterior displacement is more common than anterior displacement, a phenomenon also seen in our case series.^20^ Interestingly, the severity of displacement was substantially low at 1.5 mm, which is in conjunction with the findings of Reinhold et al. The authors found a displacement of the odontoid fracture of 1.8 mm in the conservative group, while patients opted for surgery had higher degrees of displacement (4.7 mm).^20^ Previous evidence supports that the severity of displacement might be a significant confounder when deciding between a surgical and non-surgical approach. Operative management is recommended in older patients, in cases of posterior displacement of the fracture, and when there is displacement of >4-6 mm.^21^ Our dataset could not detect a significant influence of the trauma mechanism on fracture displacement. We therefore believe that, rather than high vs low-energy trauma, the degree of displacement should be the prime parameter considered when evaluating patients for surgical therapy.

### Strengths and limitations

This study is the first to systematically examine potential predictors such as established degeneration patterns and radiological measurements in older patients suffering from a type II odontoid fracture after HET and LET. The main strength is that the power of our results was over 80%, thus supporting their generalizability. However, some limitations do exist. First, we examined a relatively small cohort. However, as this study comprised older individuals suffering from HET but also LET, a patient group that has played only a marginal role in previously published reports, we believe that our findings offer a powerful tool to present a real-world picture of the disease and will contribute to decision making. In addition, one might deem that such radiological measurements depend on the examiner. To verify the accuracy and fidelity of the results, we performed an additional analysis to determine the rate of interobserver agreement, which showed excellent agreement in describing all parameters. As this is a retrospective study, selection bias may have been present. However, according to the power analysis, although a relatively low number of patients was examined, our findings were meaningful. Given the displacement of the odontoid fracture, one might deem that the angles of cervical alignment were estimated higher. However, since the amount of displacement was relatively low, we believe that the presented parameters of cervical alignment reflect a real picture when considering older patients. A further limitation might be that the position of the patients during the examination (supine/upright) was not considered as a potential confounder. Since no dynamic X-rays were performed to diagnose the atlantoaxial instability and dislocation, our findings concerning the ADI should be regarded with caution.

## Conclusions

Our findings showed significant higher rates of osteoporotic and degenerative changes in patients suffering from low-energy trauma. Furthermore, the C2-C7 SVA, C0-C2, and C1-C2 angles were significantly higher in the LET group.
